# Assessing the role of family level variation and heat shock gene expression in the thermal stress response of the mosquito *Aedes aegypti*

**DOI:** 10.1098/rstb.2022.0011

**Published:** 2023-03-27

**Authors:** Fhallon Ware-Gilmore, Mario Novelo, Carla M. Sgrò, Matthew D. Hall, Elizabeth A. McGraw

**Affiliations:** ^1^ Department of Entomology, The Pennsylvania State University, University Park, PA 16802, USA; ^2^ The Center for Infectious Disease Dynamics, The Pennsylvania State University, University Park, PA 16802, USA; ^3^ Department of Biology, The Pennsylvania State University, University Park, PA 16802, USA; ^4^ School of Biological Sciences, Monash University, Melbourne, Victoria 3800, Australia

**Keywords:** climate warming, thermal tolerance, adaptation, heat shock, heritability

## Abstract

The geographical range of the mosquito vector for many human disease-causing viruses, *Aedes aegypti*, is expanding, in part owing to changing climate. The capacity of this species to adapt to thermal stress will affect its future distributions. It is unclear how much heritable genetic variation may affect the upper thermal limits of mosquito populations over the long term. Nor are the genetic pathways that confer thermal tolerance fully understood. In the short term, cells induce a plastic, protective response known as 'heat shock'. Using a physiological ‘knockdown’ assay, we investigated mosquito thermal tolerance to characterize the genetic architecture of the trait. While families representing the extreme ends of the distribution for knockdown time differed from one another, the trait exhibited low but non-zero broad-sense heritability. We then explored whether families representing thermal performance extremes differed in their heat shock response by measuring gene expression of heat shock protein-encoding genes *Hsp26, Hsp83* and *Hsp70.* Contrary to prediction, the families with higher thermal tolerance demonstrated less *Hsp* expression. This pattern may indicate that other mechanisms of heat tolerance, rather than heat shock, may underpin the stress response, and the costly production of HSPs may instead signal poor adaptation.

This article is part of the theme issue ‘Infectious disease ecology and evolution in a changing world’.

## 1. Introduction

Dengue fever is the world's most prevalent arboviral disease [1]. In the last 50 years, dengue incidence has increased drastically, with over 390 million infections thought to occur annually [2,3]. Urbanization and warming temperatures are associated with the spread of the virus's main vectors, *Aedes aegypti* and *Aedes albopictus* [2,4,5]. As the Earth's temperature changes, the concern is that *Aedes* mosquitoes and the viruses they transmit will spread to wider latitudes and higher altitudes, and transmission seasons will lengthen in current endemic areas [6]. This range expansion has already been seen in *Ae. albopictus*, the more temperate associated vector of the two species [7]. In addition to dengue virus (DENV), these two vector species are also responsible for transmitting Zika, chikungunya and yellow fever viruses [2], and hence changing vector distributions may have widespread effects on human health. Understanding the mechanisms underpinning mosquito thermal tolerance will allow us to better assess the future effect climate change will have on mosquito-borne disease transmission [8].

Mosquitoes have a complex lifecycle that includes an aquatic and terrestrial stage. Mosquitoes are particularly sensitive to environmental warming because of the effect temperature has on their physiological function [9]. As ectotherms, mosquito thermal regulation is dependent entirely on ambient temperature, and hence it is a major driver for organismal adaptation, influencing every level of biological organization from molecular kinetics to species distributions [10–12]. Mosquitoes may respond to environmental warming through short-term coping and avoidance mechanisms supported through phenotypic plasticity, including changes in behavioural regulation (i.e. shifts in resting and biting behaviour), quiescence, use of microhabitats, and differential oviposition site selection. In the long term, mosquitoes may cope with climate change through adaptive evolutionary change, which could result in shifts in their thermal tolerance (i.e. the ability to survive a potentially lethal exposure to extreme thermal stress) [13–16].

Temperature has a strong nonlinear effect on fitness-relevant life-history traits, including survival and fecundity [6,7], and therefore has additional influences on mosquito-borne disease dynamics. It also affects life cycles, physiology, competence for pathogens, and animal behaviour [10,17–21]. Several recently published studies have generated predictive maps of both mosquitoes and disease under patterns of future climate warming [4,7,17]. They predict that in some geographical regions, where temperatures exceed critical upper and lower thermal limits (i.e. movement ceases/death ensues), the risk of transmission will decrease, whereas regions currently below thermal optima (i.e. physiologically ideal temperatures) may experience increased transmission [7,17,22]. These predictive models fail to include the potential for mosquitoes to evolve and adapt to a changing climate. This is because we have a poor understanding of the genetic basis of thermal tolerance and how variation for the trait is distributed across current global populations [16].

Few studies have measured the levels of heritable genetic variation in thermal tolerance in mosquito populations [16], but there have been several that have measured phenotypic differences in tolerance between populations [14,23–27]. Higher heritability in thermal tolerance would allow more rapid evolution and adaptation of populations in response to changing temperatures [28–30]. Some empirical and theoretical work suggests that there is a limited capacity for heat tolerance to evolve and that heritability for environmentally dependent traits like thermal tolerance is expected to be low [28,30–32]. However, there are other studies that report significant levels of heritability for this trait, including, for example, two species of rainforest-dwelling *Drosophila* [32,33], implying that there is potential for this trait to evolve.

Unlike in *Drosophila*, the potential mechanisms for adaptation to climate change in mosquitoes are less well characterized. Addressing the potential for evolutionary adaptation will require an understanding of the factors and mechanisms that may enable persistence in warming temperatures, including: coping with thermally stressful conditions through phenotypic plasticity, heat tolerance through evolutionary adaption, and shifts in range distribution in response to potential environmental suitability [6,15,16]. A recent review by Couper *et al.* [16] synthesizes the current understanding of mosquito adaptive potential for thermal stress and highlights key knowledge gaps that can only be addressed with additional empirical studies. Outstanding questions include the relative importance of genes versus environment in shaping thermal tolerance, the identification of genetic pathways that underpin thermal tolerance traits, the amount of variation in evolutionary potential for thermal tolerance, and how ‘thermal performance’ (i.e. mosquito specific fitness traits quantified over a range of ambient environmental temperatures) varies across global landscapes [6,16,34,35] and through time (i.e. seasonality) [36].

In typical and warming conditions, mosquitoes will encounter various environmental stressors, including heat stress, that disturb cell homeostasis and require biochemical and physiological adaptions to ensure survival [9,37]. Mosquitoes are distributed across heterogeneous environments, and in these habitats they face a range of temperatures that can fluctuate daily and seasonally [10,18,38]. At the molecular level, stressful temperatures can disrupt protein function, which can lead to impairment of cell function [39]. To cope with thermal and other stresses (e.g. infection, UV, desiccation, oxidative stress), cells induce a ‘heat shock response’ (HSR) [40].

The HSR is a series of cellular responses to physiological and environmental stressors that result in the production of heat shock proteins (HSPs). HSR is transcriptionally regulated by heat shock transcription factors (HSFs), which are vital for varying physiological functions and protecting cells against proteotoxic stress. HSPs play a major role as molecular chaperones, assisting other proteins in keeping their native conformation and protecting cells from possible damage caused by misfolding and aggregation [41].

*Hsp* gene expression can play an adaptive role in response to environmental stress during periods of rare and unexpected thermally stressful exposures [42,43]. In mosquitoes, *Hsp* expression is upregulated in *Ae. aegypti* larvae and pupae following exposure to heat shock [44]. The relationship between inducible *Hsp*s and the response to heat is not always predictable or clear. In various species of *Drosophila*, it was shown that expression of *Hsp70* was lower in lines that had been previously and continuously exposed to severe heat stress [45]. The interpretation was that the cost of continuous *Hsp* expression may be too high, and that adaption to thermal stress was therefore being achieved through other means [46,47]. There is also an interesting connection between the HSP chaperone network and the DENV life cycle [48]. Several of these chaperones appear to be co-opted by DENV to assist with its replication inside mosquito cells [49,50]. Specifically, HSP90 and HSP70 have both been shown to assist with infection of human and mosquito cells for multiple mosquito-transmitted viruses [36–38,51,52].

In *Drosophila* and other invertebrates, a range of physiological assays are routinely used in the laboratory to quantify thermal tolerance [53,54]. Insect performance in these assays has been shown to predict species distributions in the wild [55]. Previously, we used a similar heat knockdown (KD) assay in *Ae. aegypti* to ascertain the effects of microbial infection on mosquito thermal tolerance [56]. Here we have paired the same assay with mosquitoes bred from a single population in a modified full-sib design to estimate the heritability of thermal tolerance. We have also examined the expression of several *Hsp* genes in the mosquito families exhibiting differences in mean KD time. Understanding the mosquito's potential to adapt to a changing climate is necessary for developing accurate predictive models for mosquito- and other vector-borne disease distributions in the future.

## 2. Methods

### (a) Mosquito lines and rearing

*Aedes aegypti* collected previously from Monterrey, Mexico, were reared in the laboratory for five generations (F_5_) before experimentation to allow expansion of the population. The laboratory population was founded based on thousands of eggs collected over several months across an urban trap grid. Larvae were reared at low densities and supplemented with fish food (Tetramin, Tetra). Adults were also reared at low density (200–250 per 45 cm square cage) and fed 10% sucrose daily ad libitum. All developmental stages were maintained at 26 ± 0.5°C with 65% relative humidity with a 12 h : 12 h light : dark cycle. Eggs were obtained each generation after blood-feeding mated females on human blood (BioIVT) using an artificial feeder (Hemotek).

### (b) Breeding design

**Figure 1.**
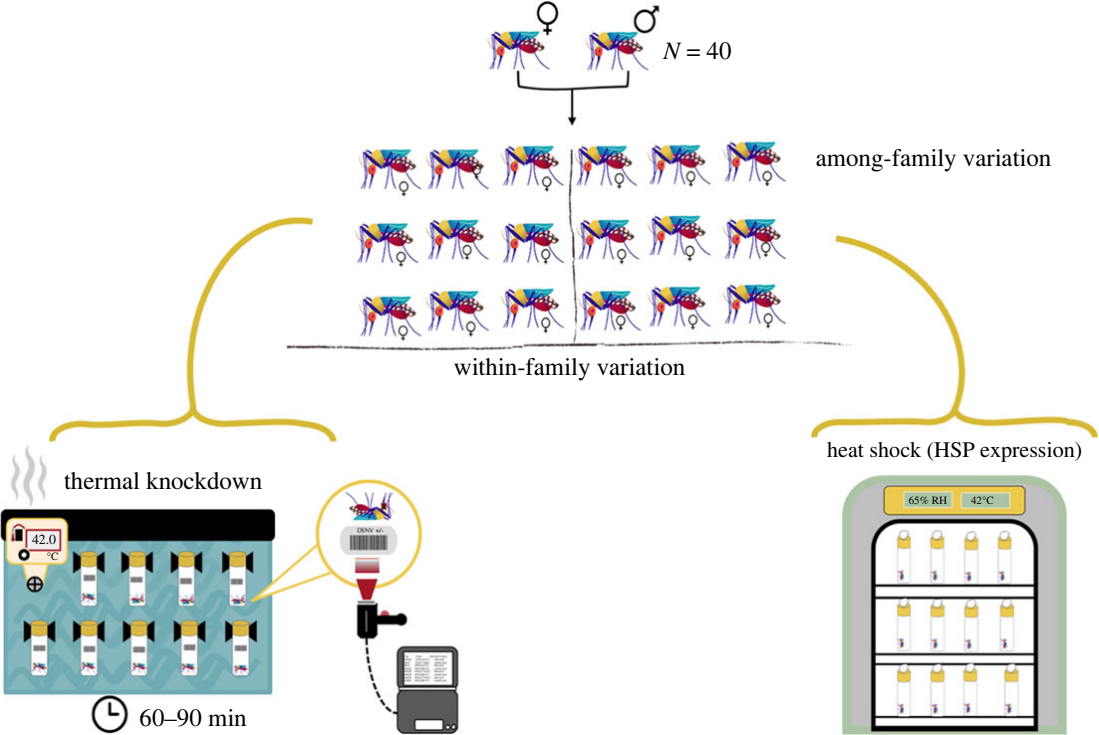
To test variation in mosquito thermal sensitivity, we submerged glass vials containing mosquitoes in a tank of water heated to 42°C, representing the upper critical thermal limit for the mosquitoes as determined by pilot assay. We then monitored the time it took for mosquitoes to become immobilized, or the ‘knockdown’ (KD) time, using a barcode scanner. For the other half of this design, we heat-shocked mosquitoes from the same families as the aforementioned mosquitoes measured for KD for 15 min at 42°C to induce stress-based expression. We then examined the expression of key heat shock genes (*Hsp*s) in selected families. (Online version in colour.)

We carried out a modified full-sib breeding design [57] to assess the role of genetic variation in KD time. From a large mated blood-fed population, we set up approximately 400 isolated females in small urine specimen cups (180 ml) and collected their eggs on filter papers. The eggs from these individual females were then dried and stored. For females that laid more than 60 eggs (figure 1), the offspring were hatched and raised to adulthood, mated, and females split into two cohorts for subsequent experimentation. Each cohort (two per family) was blood-fed 5 days post eclosion, and any unfed mosquitoes were removed the next day by sorting on ice. Each cohort was housed together until day 5 post-feeding and maintained on 10% sucrose, at which point the cohorts were split randomly and assigned to either the KD or heat shock assay. For the knockdown assay, individual mosquitoes were placed into 40 ml glass vials [56], and for the heat shock assay, mosquitoes were placed in 32 oz paper cartons in groups of five or six.

### (c) Thermal knockdown assay

As in Ware-Gilmore *et al.* 2021 [56], thermal tolerance of mosquitoes was measured using a static heat shock assay (figure 1) based on previous work in *Drosophila* [58], *Daphnia* [54] and mosquitoes [56]. Experiments were carried out at 42°C, a temperature deliberately selected to force mosquitoes beyond their upper thermal limit (greater than 95% death) within 90 min (also as per [56]). The length of the assay allowed us to assess sufficient replicate KD assays in a single day. Assays were performed 5 days post blood feed (DPI). Just prior to each assay, the mesh lids of the glass vials were replaced with solid plastic lids. The vials containing the mosquitoes were then attached to a vertical board in groups of 40 via anchored clips. The board with vials was immersed in a water bath at 42°C, and mosquitoes were allowed a 60-second acclimation period before recordings began. Mosquitoes were then monitored visually for immobility and time to thermal KD, scored using Brady labels and a TriCoder Scanner (Worth Data Inc., Santa Cruz, California). Immobility was confirmed by tapping on the vial. In total, we assayed 40 families, each represented by seven individual females.

### (d) Heat shock assay (acute heat stress)

To test how high temperatures affect gene expression, we exposed adult mosquitoes in their family groups to extreme heat stress (42°C) and then collected whole mosquitoes for RNA extraction and subsequent PCR. We also included expression of the HSPs in age-matched mosquitoes that were not exposed to heat from the base-rearing population used to create the families. While this control does not capture any unique effects of the breeding design on expression, it does provide context for uninduced *Hsp* gene expression levels in the mosquito line. Adult females representing the 40 families were exposed to 42°C in an environmental chamber in groups of five or six per family for 15 min. This method and length of exposure were based on previous heat shock studies in *Drosophila* [59] and in *Ae. aegypti* pupae and larvae [60]. After the initial heat shock, mosquitoes were snap-frozen and stored at −80°C until extraction. We subsequently added 300 µl of Trizol reagent (Invitrogen) containing a 2 mm glass bead, and samples were homogenized using a Bead Ruptor Elite (OMNI International, Kennesaw, Georgia). RNA extraction was then performed according to the manufacturer's instructions [61].

### (e) Expression of heat shock protein-encoding genes in response to heat stress

cDNA synthesis and gene expression were performed on a LightCycler 480 (Roche) using qScript One-step SYBR Green qRT-PCR according to the manufacturer's protocol. cDNA was produced at 50°C for 5 min, followed by a *Taq* inactivation at 95°C for 2 min. The PCR amplification conditions were as follows: 45 cycles at 95°C for 3 s and 60°C for 30 s, followed by a melt curve analysis using a LightCycler 480 Real-Time PCR system (Roche). All *C*_t_ values were normalized to the housekeeping gene, and expression was calculated using the 2^−ΔΔ*C*_t_^ method [62]. *Hsp* gene-specific primers and *RPS17* (housekeeping gene) were based on previously published studies for *Ae. aegypti* (electronic supplementary material, table S1) [44,63]. Hsp26 and Hsp83 have been identified as critical markers of thermal tolerance and act as possible proteins essential in enhancing the survival of immature stages of *Ae. aegypti* larvae and protecting them from the full burden of thermal stress [44]. Hsp70 is another robust heat-inducible protein shown to be upregulated in *Ae. aegypti* during consumption of a hot blood meal, and that also plays a major role in dengue virus infection dynamics [50,64].

### (f) Analysis of genetic variance

Genetic variance and subsequent broad-sense heritability (*H^2^*) for the focal trait (KD time) was estimated using a modified full-sib breeding design and the following random-effects linear model:

$$z_{ijk}=b_{i}+f_{j}+\varepsilon_{ijk},$$

where *z_ijk_* is the trait value for the *k*th female, *b_i_* is the fixed effect of the *i*th experimental block, *f_j_* is the random effect of the *j*th family and *ε_ijk_* the unexplained error. To test whether genetic variance was greater than zero, this model was compared with a simplified model that had the family term omitted. The resulting probability level was then halved as variance components are constrained to be greater than zero. Broad-sense heritability was calculated as twice the genetic variance (*σ* family) divided by the total phenotypic variance (*σ*_family_ + *σ*_error_). Estimates of broad-sense heritability are indicated by one-tailed *p*-values. All models were fitted using R (v. 3.3.3; R Development Core Team) and the *Sommer* package (2016).

## 3. Results

### (a) Heritability of thermal response

By immersing glass vials containing individual mosquitoes in heated water near their upper thermal limit (42°C) and measuring time to immobility (figure 1), we have obtained an estimate of thermal sensitivity. We found heritability for the trait to be low and slightly greater than zero (*H*^2^ = 0.14, s.e. = 0.09, d.f. = 1, *p* = 0.028). Furthermore, variation was seen in KD time across the distribution, and when we compared the five families from the ends of the distribution (electronic supplementary material, figure S1), the means were significantly different from one another (unpaired *t*-test, *t* = 6.48, d.f. = 68, *p* < 0.0001). Families at the lower end of the distribution had a KD time that was, on average, 2.6-fold faster than families at the upper end. This led us to investigate and compare the *Hsp* gene expression in the families from the extreme ends to assess the potential role of this plastic response in the phenotypic differences in KD time.

### (b) Effect of thermal stress on heat shock protein gene expression levels

**Figure 2.**
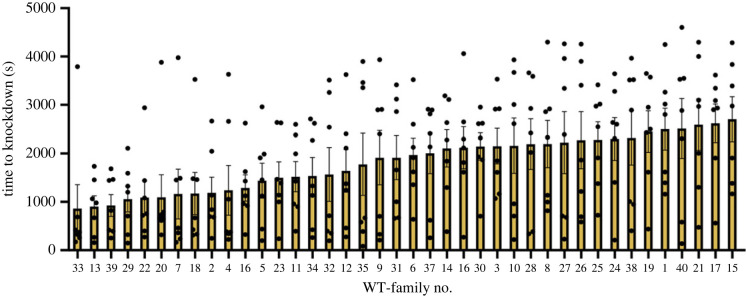
Mean ± s.e. time to knockdown for 40 mosquito families. Heritability was low but significantly different from zero (*H*^2^ = 0.14, s.e. = 0.09, d.f. = 1, *p* = 0.028). WT, wildtype. (Online version in colour.)

HSP genes *Hsp26*, *Hsp83* and *Hsp Cognate 70* are commonly expressed in response to thermal stress in *Ae. aegypti* and *Ae. albopictus* [63]*. Hsp* gene expression was measured in the sisters of females from the same families tested in the KD assay, following exposure to 42°C for 15 min. Only the five fastest and five slowest families were exposed, and genes were subsequently assessed for *Hsp* expression. A control group of age-matched mosquitoes in groups of twenty were collected from the original stock breeding population for the family design and served as controls for baseline expression of heat shock genes. Expression in families at the extreme end of thermal KD was compared with one another and with baseline (uninduced expression). Using log-transformed data, we used a mixed-effect model with the fixed-effect factor of ‘KD time’ (long time to KD ('long KD'), short time to KD ('short KD'), control) and ‘family’ (replicate) as a random effect (figure 2).

**Figure 3.**
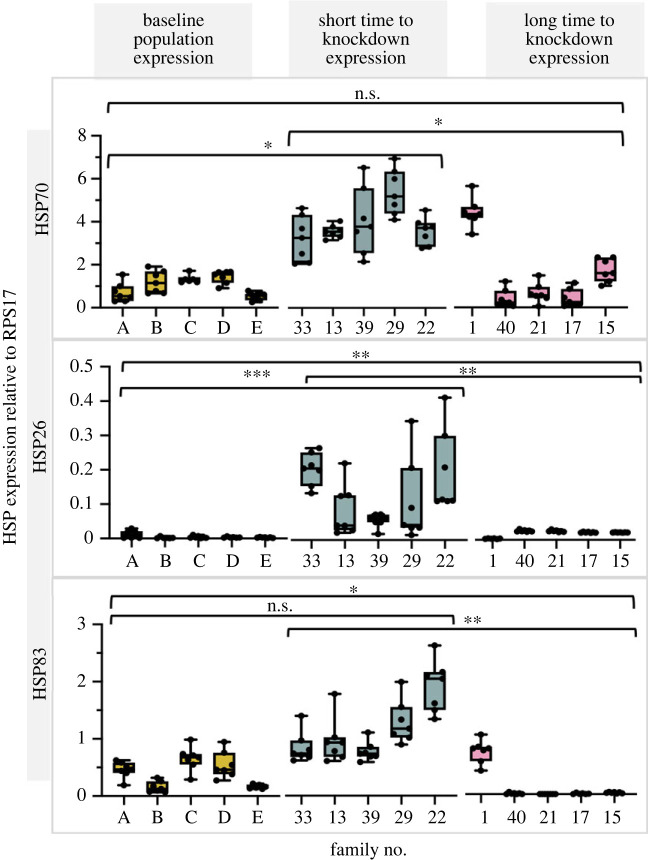
Minimum, maximum and mean ± s.e. expression for *Hsp* *70*, *Hsp26* and *Hsp83* relative to *RPS17* for five families each representing (i) a baseline population (no heat-exposed control), (ii) short (fast) KD time families and (iii) long (slow) time to KD families. *p < 0.05, **p < 0.01, ***p < 0.001. (Online version in colour.)

For *Hsp70* we saw a significant effect of KD (*χ*^2^ = 13.20, d.f. = 2, *p* < 0.001, family variance = 0.52), which was likely driven by significantly higher levels of expression in families with a short KD time. Specifically, short KD families had *ca* 4.8- and 2.7-fold higher mean expression than either the control or long time to KD group, respectively (figure 3). Control group and long KD family gene expression did not differ from one another (*p* = 0.93). However, control versus short KD groups were significantly different from each other (*p* = 0.02). In *Hsp26* we also saw a significant effect of KD group type (*χ*^2^ = 82.39, d.f. = 2, *p* < 0.001, family variance = 0.20). As for *Hsp70*, differences were driven by significantly higher expression levels in families with a short time to KD. The short KD group had 2.9- and 38-fold higher mean expression than long KD and control groups, respectively (figure 3). For *Hsp*83, we saw a significant effect of KD group (*χ*^2^ = 19.88, d.f. = 2, *p* < 0.001, family variance = 3.06). *Post hoc* comparisons (electronic supplementary material, figure S3) revealed that this was driven by significantly lower levels of expression in the families with long time to KD, relative to the other treatments (figure 3). Additionally, for *Hsp83*, short KD families differed significantly (*p* < 0.001) from long families and had a sevenfold higher mean expression than the long KD groups. The control families, on average, exhibited 2.8-fold higher expression than long KD families. In comparison, short families did not differ compared with controls (*p* = 0.56). From these results, we see that after exposure to heat stress, families with higher (longer) thermal tolerance demonstrated less expression for all three *Hsp* genes than families with low (short) resistance to heat.

## 4. Discussion

A substantial body of work exists describing the impact of average increases in the ambient temperature on mosquito fitness [6,10,65], but the consequences of dealing with extreme thermal events remain overlooked, despite the increasing frequency of these events (e.g. heatwaves, floods, droughts). For this reason, we chose to focus on mosquito thermal tolerance to an extreme temperatures representing their upper thermal limit. The upper thermal limits of performance are frequently either assumed or underestimated, as per [66], making potential predictions about tolerance in warming environments inaccurate. Having a greater focus on the upper ends of thermal performance curves may allow better estimates of ectotherm success in variable climate gradients [8,66].

To our knowledge, this is the first study to measure the heritability of thermal tolerance in mosquitoes. We revealed a low but non-zero broad-sense heritability (*H*^2^) for heat tolerance, despite finding evidence of phenotypic variation in the trait. This indicates that our study population likely has little capacity for thermal adaptation as measured by the KD assay. Mosquito populations with a greater proportion of phenotypic variation for thermal tolerance, which is attributed to genetic variation, may be better placed to adapt to climate change via natural selection [14,16,67]. Our findings are not completely surprising given previous studies in other organisms that have suggested heritability is generally low for complex and environmentally dependent thermal traits (i.e. heat tolerance and behavioural thermoregulation) [32,51]. Studies in *Drosophila* have also shown a low heritability, specifically for similar measures of KD [52].

Estimates of heritability (both broad *H*^2^ and narrow *h*^2^ sense) for heat tolerance can be environment-dependent, affected by either the environmental context or prior thermal history [33]. For example, adult rainforest-dwelling *Drosophila* were reared under two distinct thermal regimes simulating an increase in seasonal temperature of the Australian tropics [33]. Heritability for the upper thermal limits was only significantly different from zero for those flies that experienced the extreme summer thermal regimes and not for those individuals in winter or constant temperatures. The researchers in this study deduced that this was likely caused by increased additive genetic variation for thermal tolerance under the summer regime, a high-stress novel environment. Additive genetic variance may increase in novel environments because of selection acting on the percentage of rare alleles in the flies' home environment [33].

Our finding of non-zero heritability (0.14 ± 0.09, *p* = 0.02) for thermal tolerance suggests that there is some potential flexibility in mosquito heat tolerance that may buffer the effects of increasing temperatures predicted under climate change. Populations exhibiting substantially larger levels of heritability in thermal tolerance may be better able to respond via natural selection [30]. The low heritability in our study suggests a greater impact of environmental or stochastic factors in determining thermal sensitivity and KD time [11]. Our data suggest that given exposure to thermal extremes, our population would not likely survive by ‘evolutionary rescue’ and would, instead, go locally extinct. However, it is important to note that adaption to potentially lethal stress, expected in warming environments, will also depend on phenotypic variation within and between populations and additional patterns of demographic stochasticity, genetic correlation and the rate of environmental warming [34]. Constraints in organismal evolvability for upper thermal limits will be affected by current use and overlap of climatic niches, where it has been suggested that evolution in thermal tolerance for cold-adapted species may be more likely than for species whose niches are already close to their upper thermal limit [68]. Species near their upper thermal threshold may be more constrained in their ability to evolve physiological tolerances to increased temperatures and more likely impacted by the effects of temperature exceeding their current temperature limits [68].

A range of factors may have affected our estimates of thermal tolerance and its heritability. First, we studied only a single inbred population with a unique genetic composition, history of adaptation, and exposure to stochastic processes. Our methodology, including our KD assay, may also have affected the estimate of heritability. For example, static assays with different durations of exposure or ramping assays have been shown to provide different estimates of heritability for thermal tolerance in the same population [69]. A study of the evolutionary potential for heat tolerance in *Drosophila subobscura* found that heritability was higher in static versus ramping assays. The nature of the thermal stress experienced during ramping assays may lead to underestimating any genetic associations [69]. Despite these differences, it has been suggested that all measures of thermal tolerance will contribute to the evolutionary response to selection from a multivariate perspective [52,70]. Last, we measured the broad sense heritability (*H*^2^)—or the estimate of phenotypic variation due to genes that may also include dominance and epistatic effects. In the future, artificial selection approaches on thermal tolerance may be used to estimate the narrow sense heritability (*h*^2^)—or the proportion of the variation due to additive genetic effects and, therefore, the population's capacity to evolve.

The phenotypic differences in families representing the extremes in KD time could be due to either genetic differences that were not a common feature of the total population (low heritability) or environmentally affected changes. The heat shock response (HSR) is one example of a plastic and environmentally determined genetic response. Our study also investigated whether heat shock gene expression may mirror the phenotypic differences between families in thermal tolerance. This plastic response could vary by family if they experienced heat shock differences in their environment, including other stressors, although we sought to rear mosquitoes under uniform conditions. Increased heat shock gene expression was previously shown to improve survival following heat stress for *Hsp26* and *Hsp83* in immature mosquito stages and contribute to dehydration tolerance in adults for *Hsp70* [71]. The low expression of *Hsp26* seen here across all treatment groups may reflect the specificity of the gene's expression in the larval stages. We predicted that families exhibiting greater thermal tolerance may also have higher gene expression, given the protective effect of HSPs [44,46,63]. This prediction is consistent with a study in *Ae. aegypti* larvae demonstrating an increase in *Hsp* expression post-exposure to a thermal extreme and the fact that *Hsp* expression levels have been linked to species distributions across geographical clines [42,43]. Instead, we saw the opposite relationship, with higher expression in the families with reduced thermal tolerance. This pattern led us to suggest that other mechanisms of heat tolerance may be more important for the insect in response to increased temperatures and, interestingly, that increased expression of the HSR in our extreme families may, instead, be a signal of poor thermal adaptation.

*Hsp* expression has costs and benefits depending on the length and degree of elevated expression [72]. There are various lines of evidence examining the consequences of *Hsp* expression, and in *Drosophila,* small to moderate increases in *Hsp70* can account for increased thermotolerance, while large increases in expression have accounted for reduced tolerance and developmental failure and mortality [40,41,73]. Additionally, at a high concentration HSPs are toxic, as they can interfere with cellular function, and the synthesis and degradation of these also serve as an energetic drain for cellular reserves [73]. Furthermore, the accumulation of HSP's has been associated with long-term harm to organismal growth, development, and fecundity [46,74,75]. This would suggest that the intermediate expression of HSPs might be favoured by natural selection [76–78]. Control in moderating *Hsp* levels is suggested to have evolved through negative and positive tradeoffs related to tolerance to thermal stress and longer-term development and fecundity [41,72,74] which has been suggested in previous work looking at the role of HSP expression in insect thermotolerance [79,80]. The impact and limitations in the expression of these genes have led us to believe that because *Hsp* expression can be costly, in most contexts, it is likely that species that experience broad thermal ranges may be able to better conserve their *Hsp* expression, and use other aspects of the insect HSR instead of *Hsp* upregulation [11,12]. For such populations, a constant or highly reactive HSR may indicate poor adaptation to heat stress [73,74].

We suggest that other mechanisms, beyond *Hsp* expression, must explain differences in our KD performance [59]. To cope with high temperatures, insects use various mechanisms that allow them to acclimate to thermal conditions [11,12]. Maximum thermal tolerance in an individual depends on various physiological mechanisms and the functioning of several systems, including respiratory, circulatory and nervous [11,12,81]. The insect nervous system is essential in the perception of temperature and the triggering of behavioural and physiological responses to high temperatures. Metabolic rates tend to increase during thermally stressful conditions, although insects can counter this shift via protein phosphorylation that suppresses downstream metabolic loci [11,82]. Furthermore, thermoregulation is another strategy insects employ, occurring through endothermy or evaporative cooling [83]. Last, the HSR itself triggers the synthesis of neuroendocrine factors and biogenic amines. These agents are suspected to drive some of the most rapid changes in heat tolerance in insects [11,84]. Some biogenic amines, including epinephrin and octopamine regulate Na^+^/K^+^-ATPase activity [85]. In orthopterans, this regulation in Na^+^/K^+^-ATPase activity is thermo-protective, and increased octopamine specifically assists with recovery from heat and with overall tolerance [86]. In *Drosophila*, mutations altering biogenic amine metabolism result in reduced heat stress tolerance in females [87].

Having found little evidence of heritability for thermal tolerance in a single population, future studies should aim to measure populations spanning a variety of thermal origins, and those addressing any local adaption to thermal regimes, to test the generality of our findings [16,88]. Furthermore, we also suggest that further research aim to examine the role of heritability in additional traits related to climate sensitivity and mosquito fitness. These studies may also wish to compare different methodologies for estimating thermal tolerance [53]. Similarly, while we conclude that increased *Hsp* expression may be a sign of poor adaption to thermal stress in our population, this may result from its own unique history of adaptation. Future studies may wish to fully explore the benefits of *Hsp* expression in mosquitoes relative to fitness costs to understand under what conditions the HSR is likely to be an effective strategy or when other mechanisms are used.

## Data Availability

All raw data for this study are available at Figshare: https://doi.org/10.6084/m9.figshare.19092110 [89]. The data are also provided in the electronic supplementary material [90].
